# Speech to noise ratio improvement induces nonlinear parietal phase synchrony in hearing aid users

**DOI:** 10.3389/fnins.2022.932959

**Published:** 2022-08-09

**Authors:** Payam Shahsavari Baboukani, Carina Graversen, Emina Alickovic, Jan Østergaard

**Affiliations:** ^1^Department of Electronic Systems, Aalborg University, Aalborg, Denmark; ^2^Integrative Neuroscience, Department of Health Science and Technology, Aalborg University, Aalborg, Denmark; ^3^Department of Health Science and Technology, Center for Neuroplasticity and Pain (CNAP), Aalborg University, Aalborg, Denmark; ^4^Eriksholm Research Centre, Snekkersten, Denmark; ^5^Department of Electrical Engineering, Linköping University, Linköping, Sweden

**Keywords:** listening effort, electroencephalography, noise reduction, phase synchrony, local connectivity, hearing impaired

## Abstract

**Objectives:**

Comprehension of speech in adverse listening conditions is challenging for hearing-impaired (HI) individuals. Noise reduction (NR) schemes in hearing aids (HAs) have demonstrated the capability to help HI to overcome these challenges. The objective of this study was to investigate the effect of NR processing (inactive, where the NR feature was switched off, vs. active, where the NR feature was switched on) on correlates of listening effort across two different background noise levels [+3 dB signal-to-noise ratio (SNR) and +8 dB SNR] by using a phase synchrony analysis of electroencephalogram (EEG) signals.

**Design:**

The EEG was recorded while 22 HI participants fitted with HAs performed a continuous speech in noise (SiN) task in the presence of background noise and a competing talker. The phase synchrony within eight regions of interest (ROIs) and four conventional EEG bands was computed by using a multivariate phase synchrony measure.

**Results:**

The results demonstrated that the activation of NR in HAs affects the EEG phase synchrony in the parietal ROI at low SNR differently than that at high SNR. The relationship between conditions of the listening task and phase synchrony in the parietal ROI was nonlinear.

**Conclusion:**

We showed that the activation of NR schemes in HAs can non-linearly reduce correlates of listening effort as estimated by EEG-based phase synchrony. We contend that investigation of the phase synchrony within ROIs can reflect the effects of HAs in HI individuals in ecological listening conditions.

## 1. Introduction

Hearing impaired (HI) individuals often report that listening to speech in noisy environments such as competing talkers and background noise demands greater effort, which can lead to negative effects such as increased incidence of fatigue (Kramer et al., 2006; Mattys et al., 2012; Wang et al., 2018), disengagement from conversations (Jaworski and Stephens, 1998) and social withdrawal (Weinstein and Ventry, 1982). However, current measurements which are used to examine the performance of a listening task (e.g., speech reception threshold) do not typically consider the cognitive factors related to effortful listening (Sarampalis et al., 2009; Houben et al., 2013).

Pichora-Fuller et al. (2016) defined the concept of listening effort in a conceptual model as “the deliberate allocation of mental resources to overcome obstacles in goal pursuit when carrying out a task, with listening effort applying more specifically when tasks involve listening.” The obstacles include acoustic challenges experienced by the listener, which is the combination of cognitive factors (e.g., linguistic ability and memory capacity) and acoustic characteristics (e.g., level of background noise and competing talker) (Peelle, 2018). Listening effort can also be modulated by the listener's motivation (Pichora-Fuller et al., 2016; Peelle, 2018). The goal of studying listening effort is to develop a reliable measurement tool, which can be simultaneously utilized with speech recognition tests and improve the assessment of hearing disability (Paul et al., 2021) and enhance the rehabilitation strategy (Miles et al., 2017).

A wide variety of methods and tools have been used to find correlates of listening effort. This includes subjective ratings such as scales (Krueger et al., 2017) and questioners (Hart and Staveland, 1988), dual tasks (Gagne et al., 2017), and physiological measures such as pupillometry (Zekveld et al., 2018), skin conductance (Mackersie and Calderon-Moultrie, 2016), heart rate (Mackersie and Calderon-Moultrie, 2016), and neuroimaging (Paul et al., 2021). Neuroimaging measures tend to reflect changes in the brain activity underlying listening effort. In particular, electroencephalography (EEG) is becoming popular for measuring correlates of listening effort due to its non-invasiveness and high temporal resolution (Dimitrijevic et al., 2019; Seifi Ala et al., 2020; Fiedler et al., 2021; Wisniewski et al., 2021).

A diverse range of signal processing and information theoretic methods have been used to analyze the EEG and extract correlates of listening effort. Some examples include time-frequency analysis, speech tracking, and functional connectivity. The change in power in the alpha (8-12 Hz) frequency band at the parietal region (Petersen et al., 2015; Dimitrijevic et al., 2017) and theta (4-8 Hz) frequency band at the frontal region (Wisniewski et al., 2015, 2018) have been reported by using time-frequency analysis. The coherence between the speech envelope and the corresponding brain signal at the left frontal cortex in the 2–5 Hz frequency range has also been demonstrated that it can be used for predicting correlates of listening effort in speech tracking analysis (Dimitrijevic et al., 2019).

Functional connectivity describes the statistical dependencies between neural data and can give some information about how the brain functions (Bidelman et al., 2018). Functional connectivity analysis in EEG signals has been extensively used to investigate cognitive functions of auditory processing in the brain. Some examples include perceived audio quality assessment (Mehta and Kliewer, 2017) and semantic processing (Zhang et al., 2019). The effect of acoustic challenges, age-related hearing loss, and comprehension of speech on functional connectivity were also investigated in Bidelman et al. (2018, 2019), and Zhu et al. (2020), respectively.

Functional connectivity can be extracted by using several approaches such as phase synchrony (Bernarding et al., 2013), transfer entropy (Mehta and Kliewer, 2017; Baboukani et al., 2020, 2021b), and Pearson correlation (Bidelman et al., 2019). The phase of neural data tends to reflect the timing of neural activity, and phase synchrony describes the interaction between or within brain regions in the neural networks (Wöstmann et al., 2017a). Correlates of listening effort have been estimated using phase synchrony analysis. Several methods have been used to extract the phase synchrony, such as wavelet phase synchronization stability (Bernarding et al., 2010, 2013), the distribution of the mapped phase mean vector on the unit circle (Bernarding et al., 2014) and the entropy of instantaneous phase of EEG signals (Bernarding et al., 2012, 2017).

The functional connectivity within a localized region of the brain is called local connectivity. It has been utilized to classify different motor imagery movements (Baboukani et al., 2017), estimate the cognitive workload (Zarjam et al., 2013), investigate schizophrenia (Jalili et al., 2007), and Alzheimer's disease (Jalili et al., 2013). Phase synchrony has also been used to assess the local connectivity. Phase synchrony assessment across multivariate signals (or channels of EEG) in a localized region of the brain by averaging over all possible traditional bi-variate phase synchrony values (e.g., phase coherence, phase locking value) may not provide a full picture of the global synchrony within the signals (Oshima et al., 2006; Canolty et al., 2011; Omidvarnia et al., 2013; Al-Khassaweneh et al., 2016; Baboukani et al., 2019). Alternatively, multivariate measures generalized the traditional bi-variate ones to evaluate phase synchrony within multichannel data (Omidvarnia et al., 2013; Al-Khassaweneh et al., 2016; Baboukani et al., 2019). Local connectivity estimated by multivariate phase synchrony has been used in several studies (Jalili et al., 2013; Al-Khassaweneh et al., 2016; Baboukani et al., 2017). A new multivariate phase synchrony measure called circular omega complexity (COC) was recently proposed and has shown better performance than conventional multivariate phase synchrony techniques in simulated and real EEG data (Baboukani et al., 2019). Recently, we showed that local connectivity estimated by the COC measure can be used to estimate the correlates of listening effort (Baboukani et al., 2021a).

Although HI individuals in real-life encounter listening situations which involve continuous speech and long sentences, most of the studies (some exceptions include Alickovic et al., 2020, 2021; Fiedler et al., 2021) investigate the effect of NR processing and SNR when the stimuli are single words or short sentences (Bernarding et al., 2014; Dimitrijevic et al., 2017; Miles et al., 2017). However, in this study, continuous long stimuli are used in a speech in noise (SiN) task.

Modern hearing aid (HA) devices can help HI individuals through various advanced signal processing approaches (Bernarding et al., 2014, 2017; Winneke et al., 2018). In particular, noise reduction (NR) processing intends to reduce the effect of background noise and enhance the signal-to-noise ratio (SNR). It has shown the capability to free up cognitive resources for other tasks during listening and reduce the listening effort (Sarampalis et al., 2009; Ohlenforst et al., 2018). The activation of NR processing can improve speech understanding at low SNRs. Furthermore, it has been also shown that activation of the NR schemes in HAs provides a listening effort enhancement in addition to any effect associated with improved intelligibility (Ohlenforst et al., 2018). In addition to that, NR schemes can improve the performance of the HA users during a selective attention task (Alickovic et al., 2020, 2021). Alickovic et al. (2020) also showed that the improvement of selective attention tasks due to NR was different at low SNR than that at high SNR. Another study on the same data showed that NR changed correlates of listening effort estimated by pupil size differently at the two SNR values, while a time-frequency analysis of EEG signals showed no statistical change due to SNR, NR, and the interaction between them (Fiedler et al., 2021). This inspired us to replace conventional power analysis in the time-frequency domain with local connectivity estimates based on multivariate phase synchrony to investigate the effect of NR processing at two SNR values. Inspired by the results in Alickovic et al. (2020) and Fiedler et al. (2021), we hypothesized:

**H1**: The use of NR in hearing aids affects the EEG phase synchrony within localized regions of the brain (i.e., local connectivity) at low SNR differently than that at high SNR.
**H2**: The effect of the NR scheme on EEG phase synchrony within localized regions of the brain at different SNR values shows a nonlinear (inverted U-shape) trend.

## 2. Materials and methods

In this section, the EEG data utilized in this study will be briefly explained. It will be followed by the description of the COC measure and the steps required to assess local connectivity. Finally, the statistical test used in this study will be described.

### 2.1. EEG data

The EEG data of this study has been utilized for other analyses by Alickovic et al. (2020) and Fiedler et al. (2021), which focused on neural tracking and pupil dilation, respectively. The EEG analysis of listening effort recruited by Fiedler et al. (2021) was based on alpha power and did not show significant results.

#### 2.1.1. Participants

We recruited 22 (11 men), native Danish-speaking participants, with hearing loss. The stimuli used in this study were based on participants-centered language (Nicks et al., 2022) and consisted of Danish news clips of neutral content. They aged between 40 and 80 years with the mean and standard deviation (SD) ages of 69 and 11.2, respectively. The participants were experienced HA users with more than 3 months of HA usage. Participants had mild-to-moderate sensorineural hearing loss. The audiometric thresholds at 500, 1,000, 2,000, and 4,000 Hz ranged from 33 to 58 dB hearing level, with an average threshold of 45 dB hearing level. The maximum difference between the left and the right ear' averaged audiometric thresholds was less than 8 dB. The experimental procedure was approved by the ethics committee for the capital region of Denmark (journal number H-1-2011-033) and all participants signed written consent before the experiment.

#### 2.1.2. Hearing aid fitting and signal processing

All participants were fitted with identical HA models during the experiment. Two pairs of HAs were adapted for each participant: NR inactive and NR active. Rather than NR, all other signal processing settings did not change between the conditions. The Voice Aligned Compression (VAC) rationale (Le Goff, 2015) based on each individual's hearing threshold was applied to amplify the sound in both pairs of HAs. In the NR inactive condition, the omnidirectional setting was applied with an added natural slight forward effect of the pinna. In the other pairs, NR active, the combination of minimum variance distortionless response beamformer and a single channel Wiener post filter was applied before the VAC. The articulation-index-weighted SNR improvements (Ohlenforst et al., 2018) were 6.24 dB and 5.17 dB at +3 dB SNR and +8 dB SNR, respectively, for NR active than that for NR inactive (Alickovic et al., 2020; Fiedler et al., 2021).

#### 2.1.3. Experimental design

**Figure 1:**
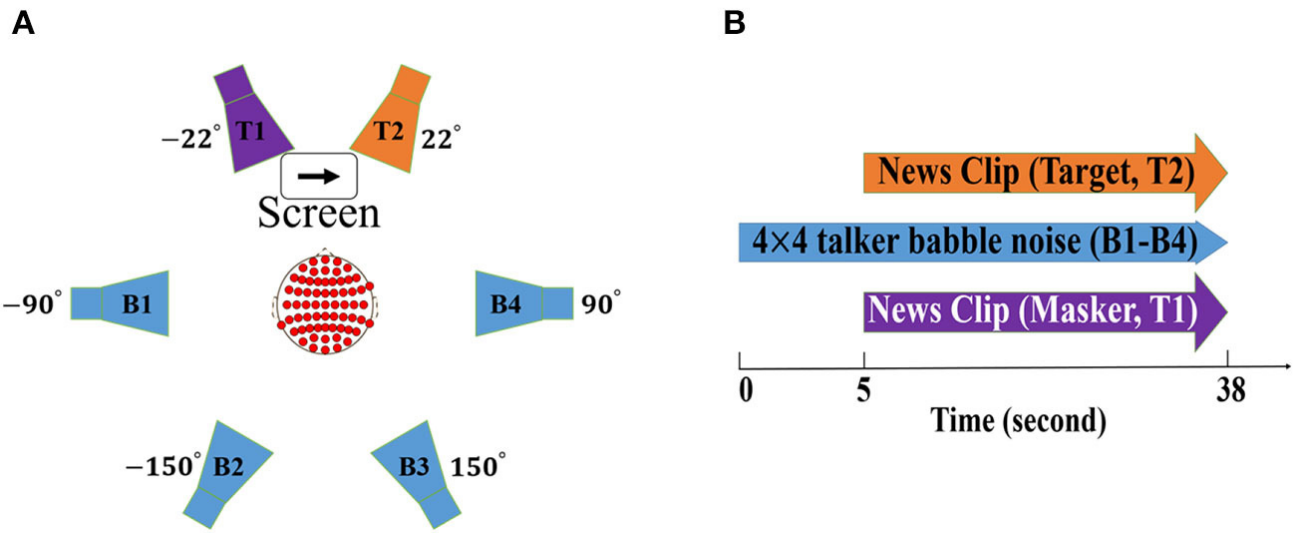
Schematic illustration of **(A)** experiment design including six loudspeakers. The target streams (colored as purple and orange) and background noise (colored as blue) were delivered by the two foreground and four background loudspeakers, respectively. The screen located in the middle of the two foreground speakers shows the to-be-attended talker (colored as orange). **(B)** trial design in which 5 s of only background noise and 33 s of simultaneous target, masker, and background noise stimuli were delivered in each trial.

The experiment took place in an acoustically shielded listening booth with controlled light conditions. Participants were seated on a chair positioned in the middle of six loudspeakers (Genelec 8040A; Genelec Oy, Iisalmi, Finland) with a distance of 1.2 m from each loudspeaker (refer to Figure 1A), two loudspeakers in the front (at ±22° azimuth) and four loudspeakers in the back (at ±90° and ±150° azimuth). Each of the background loudspeakers (B1-B4 in Figure 1A) played four-talker babble. The two foreground speakers played the target streams which were spoken by talkers of a different gender. Participants were asked to gaze at the screen positioned between the two frontal loudspeakers and were instructed to attend to one of the talkers in the foreground speakers while ignoring the other talker on the contralateral side and background noise. To-be-attended talkers (either the right or the left side) was indicated by an arrow on the screen (refer to Figure 1A).

#### 2.1.4. Stimuli

Continuous 33 s long Danish news clips of neutral content were utilized for talker streams. The organization of the location (left or right) and gender (male or female) of the target stream was randomized. Each of the four-talker babble noises delivered by the background loudspeakers consisted of four unique single talkers, two women and two men, speaking different news giving the impression of the 16 talkers active in the background.

The experiment was a 2 × 2 design: the first factor was NR (active vs. inactive) and the second factor was the SNR level (+3 vs. + 8 dB). The SNR in our setup was defined as the ratio between the signal power of the attended talker and the total signal power of the background noise, similar to that in Das et al. (2018) and Alickovic et al. (2020). In order to create real-life listening conditions at two levels of difficulty (SNR values of +3 and +8 dB), the maskers were set at either 53 or 48 dB, leading to a total of 59 or 54 dB background Sound Pressure Level (SPL). Each of the two foreground loudspeakers was always set at a fixed level of 62 dB SPL, leading to a fixed level of 65 dB for the foreground talkers.

#### 2.1.5. Procedure

A total of 84 trials were recorded for each participant, organized in a block design. For each of the four blocks (experimental conditions including +3 dB NR inactive, +3 dB NR active, +8 dB NR inactive, and +8 dB NR active), 20 trials were conducted. The remaining four trials were used for training. Each trial consisted of a short period of silence, 5 s of only background noise (delivered by background loudspeakers), and 33 s of the simultaneous target, masker, and background noise stimuli (refer to Figure 1B). After each trial, participants were asked to answer a two-choice question about the content of the attended speech which was displayed on the screen. The HAs were always removed and replaced again between the blocks. The participants were given a rest period after five trials.

#### 2.1.6. EEG data acquisition and pre-processing

The BioSemi ActiveTwo amplifier system (Biosemi, Amsterdam, Netherlands) was used to record EEG data. A total of 64 electrodes on a cap were mounted on the scalp according to the 10-20 international system. The driven right leg and common mode sense electrode were used as a reference for all other recording electrodes. The EEG data were sampled at 1,024 Hz. All electrodes were mounted by applying conductive gel to obtain a stable connection and below 50 mv offset voltage.

The pre-processing includes a 0.5 Hz high-pass filter, 95 Hz low-pass filter, and downsampling to 512 Hz. Then, The EEG channels with excessive noise were visually identified (on average, 3.1±0.8 channels were rejected) and interpolated from the surrounding clean EEG channels by using the nearest neighbor method in Fieldtrip (Oostenveld et al., 2011). The logistic Infomax independent component analysis algorithm was applied to reduce artifacts caused by eye movements, eye blinks, muscle activity, heartbeats, and single-channel noise, as implemented in Fieldtrip (Bell and Sejnowski, 1995; Delorme and Makeig, 2004). The components were visually inspected and rejected if clearly reflected as artifacts, on average, 7.9±3.6 of the components were rejected. Finally, the EEG data were epoched from 8 s before to 33 s after the onset of the target loudspeakers. The EEG data for one subject was removed from further analysis due to being excessively noisy. In addition to that, no data for one block of one participant was recorded due to technical problems.

### 2.2. Circular omega complexity

The COC measure is used in this study to extract local connectivity. This is a multivariate phase synchrony measure that was recently proposed in Baboukani et al. (2019) and was shown to perform better than the conventional measures in a particular setup. The COC assesses the level of phase dependency within multivariate signals by quantifying the dimensionality of the state-space formed by their corresponding instantaneous phases (Baboukani et al., 2019).

Estimating the instantaneous phase of a real valued mono-component discrete signal *X*∈ℝ^1 × *N*^ is the first step to calculate the COC. A Hilbert transform-based approach is commonly used whereby the instantaneous phase is estimated as Baboukani et al. (2019):


(1) $$\begin{array}{l} \phi_{X}\left[n\right]=\tan^{-1}\left(\frac{\hat{X}\left[n\right]}{X\left[n\right]}\right), \end{array}$$

where $\hat{X}\left[n\right]$ and ϕ_*X*_[*n*] are the Hilbert transform and instantaneous phase of *X*[*n*], respectively. The next step is calculating the circular correlation matrix. Considering a K-channels signal **X**∈ℝ^*K*×*N*^ and its corresponding instantaneous phase signal $\phi_{\text{X}}\in {\mathbb{R}}^{K\times N}$, the circular correlation matrix *C*^**X**^∈ℝ^*K*×*K*^ is defined as Baboukani et al. (2019):


(2) $$\begin{array}{l} C^{\text{X}}=\left[C^{\left(m,l\right)}\right], \end{array}$$

where *m, l*∈{1, 2, …*K*}. The circular correlation between the instantaneous phase ϕ_*m*_ and ϕ_*l*_ is noted by *C*^(*m, l*)^ where ϕ_*m*_ and ϕ_*l*_ are the *m*^*th*^ and *l*^*th*^ rows of **ϕ**_**X**_, respectively. The circular correlation *C*^(*m, l*)^ is given as Baboukani et al. (2019):


(3) $$\begin{array}{l} C^{\left(m,l\right)}=\frac{\overset{N}{\underset{n=1}{\sum }}\sin\left(\phi_{m}\left[n\right]-{\bar{\phi }}_{m}\right)\sin\left(\phi_{l}\left[n\right]-{\bar{\phi }}_{l}\right)}{\sqrt{\overset{N}{\underset{n=1}{\sum }}\sin^{2}\left(\phi_{n}\left[n\right]-{\bar{\phi }}_{m}\right)\sin^{2}\left(\phi_{l}\left[n\right]-{\bar{\phi }}_{l}\right)}}, \end{array}$$

where the circular mean ${\bar{\phi }}_{m}$ is given by Baboukani et al. (2019):


(4) $$\begin{array}{l} {\bar{\phi }}_{m}=\arg\left(\overset{N}{\underset{n=1}{\sum }}\exp^{i\phi_{m}\left[n\right]}\right). \end{array}$$

It was shown by Baboukani et al. (2019) that the eigenvalues of *C*^**X**^ can be used as an index for the dimensionality of the state-space formed by **ϕ**_**X**_ whereby the level of phase synchronization can be determined. The COC was then defined as Baboukani et al. (2019):


(5) $$\begin{array}{l} COC=1+\frac{\overset{K}{\underset{i=1}{\sum }}{\bar{\lambda }}_{i}\log{\bar{\lambda }}_{i}}{\log K}, \end{array}$$

where ${\bar{\lambda }}_{i}=\frac{\lambda_{m}}{\overset{K}{\underset{j=1}{\sum }}\lambda_{j}}$ and λ_*i*_; *i* = 1, …, *K* are the eigenvalues of *C*^**X**^. The COC varies between 0 and 1 where higher values show that the channels within *C*^**X**^ are more phase synchronized, which means that only fewer eigenvalues of the *C*^***X***^ are significant (Baboukani et al., 2019).

### 2.3. Local connectivity assessment

**Figure 2:**
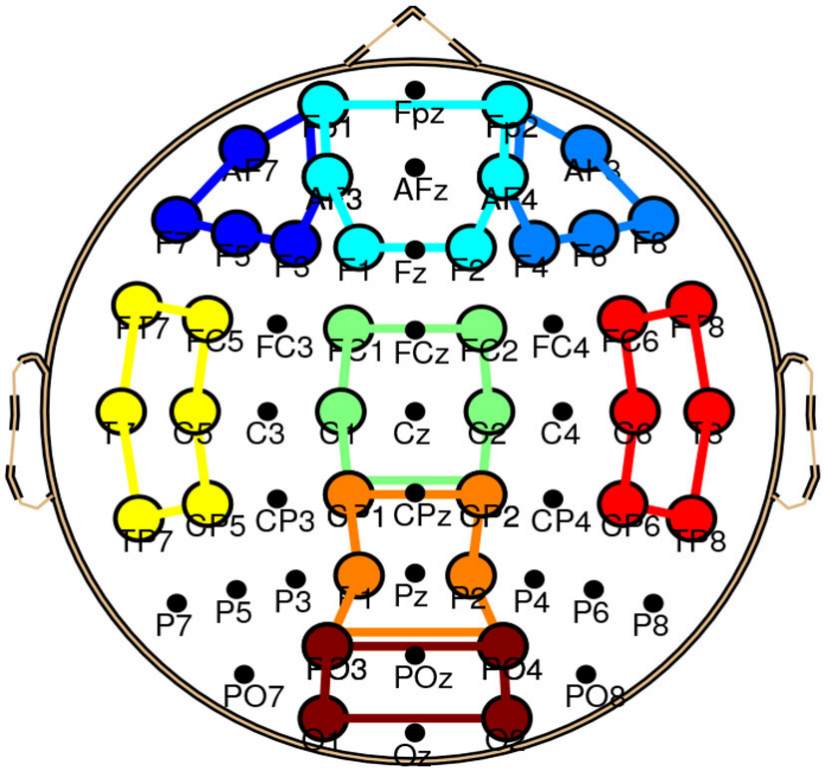
In the electrode position of ROIs, different colors show different ROIs. The lines in the figure show schematic presentations of the ROIs. The ROIs include left frontal (dark blue), frontal (aqua), right frontal (light blue), left temporal (yellow), central (green), right temporal (red), parietal (orange), and occipital (brown).

The effect of NR at two SNR values on local connectivity will be explored in this article. Listening in adverse conditions can possibly engage multiple cognitive processes such as working memory and distractor inhibition (Wisniewski et al., 2021), which can possibly change the local connectivity within different brain regions and frequency bands. We, therefore, did not restrict our analysis to one specific band or ROI and local connectivity within eight ROIs and four conventional EEG bands will be examined. The ROIs include left frontal, frontal, right frontal, left temporal, central, right temporal, parietal, and occipital, similar to that in Mehta and Kliewer (2017). Figure 2 and Table 1 show the electrodes and their corresponding positions of different ROIs, respectively ^1^. The EEG bands consist of Delta (0.5–4 Hz), Theta (4–8 Hz), Alpha (8–12.5 Hz), and Beta (12.5–25 Hz). The EEG channels were filtered by using Window-based FIR band-pass filters. Filtering to narrow frequency bands can also reduce the multi-component nature of EEG signals and can improve the estimation of the instantaneous phase signals by using the Hilbert transform technique (Boashash and A¨ıssa-El-Bey, 2018). The EEG channels were common average re-referenced to reduce the effect of volume conduction.

**Table 1:** Mapping electroencephalogram (EEG) electrodes to regions of interest (ROIs).

**ROI**	**Electrodes**	**ROI**	**Electrodes**
Left frontal	AF7, AF3, F3	Frontal	Fp1, Fp2, AF4
	F5, F7, Fp1		AF3, F1, F2
Right frontal	AF4, AF8, F8	Central	FC1, FC2, C1
	F6, F4, Fp2		CP1, C2, CP2
Left temporal	FT7, T7, TP7	Parietal	CP1, CP2, P1
	CP5, FC5, C5		P2, PO4, PO3
Right temporal	FT8, T8, TP8	Occipital	O1, O2, PO3
	CP6, FC6, C6		PO4

The local connectivity of each trial of the experiment was estimated during the time interval that frontal loudspeakers were presenting the target streams (33 s). We also omitted the first second of the delivering target streams to minimize the effects of event-related potential which led to 32 s (1 to 33 s relative to the onset of the target streams). The 32 s time span was then divided into 16 non-overlapping 2 s windows^2^. The COC was then quantified for each of the windows and the average over all windows was considered as an indicator of the strength of local connectivity. The higher the average of COC values over time windows is, the more channels within the ROI are phase synchronized. The higher phase synchrony is considered as higher local connectivity in this study. The steps required to assess local connectivity in a specific band and ROI can be summarized as follows:

S1) Band-pass filters the EEG channels in the ROI to a conventional EEG band.
S2) Estimate the instantaneous phase of the filtered channels.
S3) Extract the COC value for each of the 2 s time windows.
S4) Average the COC values corresponding to time windows.

The aforementioned steps were repeated for eight ROIs and four EEG bands leading to 32 local connectivity values for each trial.

### 2.4. Statistical test

All the statistical analysis was performed in RStudio Team (2021). In order to investigate the effect of NR at two SNR values on local connectivity (our first Hypothesis H1), two-way Linear mixed effect (LMM) ANOVA was applied by using lme4 (Bates et al., 2014) and lmerTest (Kuznetsova et al., 2017) packages. We fitted separate LMM ANOVA models for local connectivity values estimated at each ROI and band. Local connectivity values were treated as a continuous variable and normalized by using $COC_{normalized}=\frac{COC-M}{S}$, where the *M* and *S* are the mean and SD of the local connectivity values at specific band and ROI calculated over all experimental conditions and all subjects. The experiment factor NR was treated as a factor variable with two levels, inactive and active. The experiment factor SNR was also treated as a factor variable with two levels, high (+8 dB) and low (+3 dB). The local connectivity was modeled as a function of fixed factors NR, SNR, and their interaction, and the participants were treated as a random effect. The analysis was conducted based on subject-averaged local connectivity values. We will also report the results based on single trial models for the statistically significant local connectivity, in which the interaction between participants and trials was treated as a random effect.

In order to investigate our second hypothesis which is about the relationship between local connectivity and the four experimental conditions- +3 dB active, +3 dB inactive, +8 dB active, and +8 dB inactive, we applied the measured SNR improvement of the NR processing, refer to Section 2.1.2 for more details. The SNR improvements of the active NR scheme were 6.24 dB and 5.17 dB at 3 dB SNR and 8 dB SNR, respectively. This process reduces the two factors SNR and NR of the experiment to only one factor SNR, with values of 3, 8, 9.24, and 13.17 dB. Then, one-way LMM ANOVA has applied to model local connectivity as a function of fixed factor SNR which was treated as a continuous variable. Two models were used for each local connectivity: the first model included the quadratic (nonlinear) term alongside the linear term for the fixed factor SNR and the second model only consisted of the linear term. The participants were treated as a random effect. Similar to the two-way LMM ANOVA, the results based on single trial models for the statistically significant trends will be reported.

Since a series (eight ROIs and four bands leading to 32 models) of LMM ANOVA models were applied, we used the Bonferroni correction to compensate for the multiple comparisons effect. The significance levels for all the two-way and one-way LMM ANOVA models were, therefore, chosen as $\alpha =\frac{0.05}{32}=0.0016$.

## 3. Results

Participants were prompted with a two-choice question related to the content of the attended speech after each trial. They correctly answered 86% of the questions. This indicates that the participants followed the task as instructed. However, after applying a two-way LMM ANOVA on the behavioral performances, there was no statistical effect of NR, SNR, and their interaction, with the *p*-values of 0.25, 0.06, and 0.37, respectively.

To test Hypothesis H1, two-way LMM ANOVA was applied to local connectivity at each ROI and band, which modeled the normalized local connectivity as a function of fixed factors NR and SNR. Tables 2A–C summarized the *p*-values obtained from applying two-way LMM ANOVA on the average over trials for each subject local connectivity. As shown in able Table 2A, we found a significant interaction of SNR and NR on local connectivity at the parietal region alpha frequency band (will be referred to as parietal alpha hereinafter), *F*_(59.02)_ = 12.28, *p* = 0.0009. Note that, as mentioned in Section 2.1.6, the EEG data of one block for one subject was not recorded. Therefore, our data is unbalanced and the denominator degree of freedom (DF) is estimated by using Satterthwaite's method. As there is no *p*-value less than 0.0016 in Tables 2B,C, no significant main effect was found for SNR and NR. The results of applying LMM ANOVA on single trial data were in line with the average trial analysis. The interaction between SNR and NR was statistically significant at parietal alpha, *F*_(1228.5)_ = 83.59, *p* < 0.0001.

**Table 2:** *P*-values of the two-way LMM ANOVA. **(A)** P-values for interaction between two factors SNR and NR. **(B)** *P*-values of the main factor SNR. **(C)** P-values of the main factor NR. The two factors are SNR values, +3 dB and +8 dB, and NR schemes, on and off. The boldface numbers show the rejection of the null hypothesis. The significance level was Bonferroni corrected, $\alpha =\frac{0.05}{32}=0.0016$.

**(A)**								
	**Left frontal**	**Frontal**	**Right frontal**	**Central**	**Left temporal**	**Parietal**	**Right temporal**	**Occipital**
Delta	0.3083	0.9577	0.9004	0.2891	0.7109	0.0086	0.5054	0.6982
Theta	0.5986	0.9038	0.8941	0.2419	0.8460	0.0098	0.1991	0.8890
Alpha	0.5811	0.7302	0.6898	0.1370	0.5116	**0.0009**	0.04	0.6041
Beta	0.6868	0.3757	0.5041	0.2295	0.8981	0.0033	0.1544	0.7814
**(B)**								
	**Left** **frontal**	**Frontal**	**Right** **frontal**	**Central**	**Left** **temporal**	**Parietal**	**Right** **temporal**	**Occipital**
Delta	0.6430	0.1143	0.0535	0.6862	0.4565	0.7421	0.9075	0.7022
Theta	0.4882	0.0497	0.5245	0.3467	0.8811	0.4504	0.1736	0.9586
Alpha	0.6072	0.1096	0.6841	0.8241	0.8235	0.6635	0.0981	0.1719
Beta	0.2794	0.0757	0.9245	0.4805	0.6725	0.9951	0.0260	0.3742
**(C)**								
	**Left** **frontal**	**Frontal**	**Right** **frontal**	**Central**	**Left** **temporal**	**Parietal**	**Right** **temporal**	**Occipital**
Delta	0.5015	0.8633	0.7418	0.4164	0.8241	0.6195	0.6881	0.5722
Theta	0.7668	0.9642	0.5383	0.4872	0.6733	0.3703	0.8001	0.8816
Alpha	0.6963	0.7806	0.2371	0.7766	0.6075	0.4634	0.5812	0.2326
Beta	0.7135	0.5241	0.2805	0.8134	0.7648	0.7150	0.8649	0.4089

The one-way LMM ANOVA was applied to average trial data after applying the SNR improvement of the NR processing to investigate Hypothesis H2. The normalized local connectivity was modeled as a function of continuous fixed factor SNR by using two separate one-way LMM ANOVA to study the relationship between the normalized local connectivity and experiment conditions. The first model was based on including the quadratic term for the fixed factor SNR and the second model only consisted of the linear term. Table 3A shows the results of the first model in which quadratic terms alongside linear terms were included. As shown in Table 3A, the nonlinear trend between local connectivity and SNR at parietal alpha was statistically significant, *F*_(60.22)_ = 11.92, *p* = 0.0010. We found no linear relationship between the experimental conditions and local connectivity, as there is no *p*-value less than the significant level in Table 3B. The nonlinear trend between local connectivity and SNR at parietal alpha was also significant by single trial analysis, *F*_(1229.5)_ = 76.36, *p* < 0.0001.

**Table 3:** P-values of the one-way LMM ANOVA. **(A)** P-values for the quadratic term. **(B)** P-values for the linear term. The local connectivity at different ROIs and bands is independently modeled by different listening conditions. The conditions are +3 dB inactive (+3 dB), +8 dB inactive (+8 dB), +3 dB active (9.24 dB), and +8 dB active (13.17 dB). The boldface numbers show the rejection of the null hypothesis. The significance level was Bonferroni corrected, $\alpha =\frac{0.05}{32}=0.0016$.

**(A)**								
	**Left** **frontal**	**Frontal**	**Right** **frontal**	**Central**	**Left** **temporal**	**Parietal**	**Right** **temporal**	**Occipital**
Delta	0.3358	0.9458	0.9256	0.3253	0.6948	0.0094	0.4790	0.6577
Theta	0.6181	0.9998	0.9405	0.2151	0.8761	0.0129	0.2118	0.8773
Alpha	0.6061	0.7128	0.6102	0.1282	0.5417	**0.0010**	0.0593	0.5358
Beta	0.7114	0.4093	0.5719	0.2195	0.9201	0.0035	0.1618	0.8435
**(B)**								
	**Left** **frontal**	**Frontal**	**Right** **frontal**	**Central**	**Left** **temporal**	**Parietal**	**Right** **temporal**	**Occipital**
Delta	0.3699	0.2595	0.3415	0.6413	0.7490	0.4511	0.7277	0.5054
Theta	0.8699	0.2342	0.9215	0.2923	0.65551	0.6869	0.5770	0.8868
Alpha	0.9773	0.2288	0.5105	0.7980	0.7522	0.3106	0.6534	0.9599
Beta	0.3135	0.0942	0.3961	0.5875	0.9849	0.6326	0.2555	0.9071

**Figure 3:**
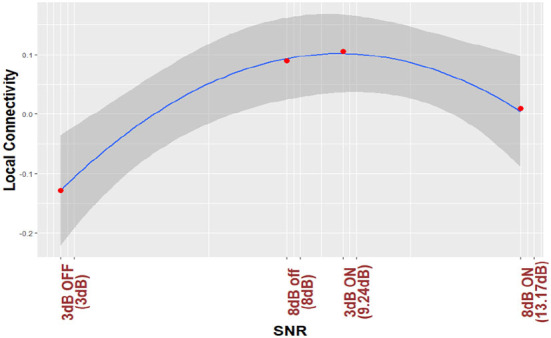
Parietal alpha local connectivity is regressed based on different listening conditions. The analysis is performed by single trial data and the red points show the average over all trials and all subjects for different listening conditions.

**Figure 4:**
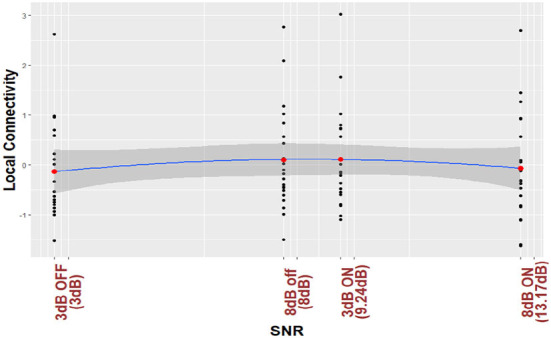
Parietal alpha local connectivity is regressed based on different listening conditions. The analysis is performed by average over trials data. The black points in the figure show the average over trials for subjects and the red points show the average over black points for different listening conditions.

For the purposes of visualization, (Figures 3, 4) plot regression line and 95% CI for the nonlinear trend between normalized local connectivity and experimental conditions at both individual and average trial analyses, respectively. The inverted U-shaped relationship shows that local connectivity at parietal alpha is higher for +3 dB active and +8 dB inactive and lower for +3 dB inactive and +8 dB active. The figures also show that NR processing at lower SNR (+3 dB) leads to an increase in the local connectivity at parietal alpha while NR processing at higher SNR (+8 dB) leads to a decrease.

## 4. Discussion

### 4.1. Summary

In a sample of 22 HIs, we studied the effect of NR processing in HAs on the EEG local connectivity during a continuous SiN task. Inspired by the results reported in Alickovic et al. (2020) and Fiedler et al. (2021), we hypothesized that the effect of NR schemes on local connectivity differs at the two SNR values, +3 dB and +8 dB, of the experiment. Consistent with our Hypothesis (H1), we found a significant interaction between the factors of the experiment, SNR and NR, at parietal alpha by using both average-trial and single-trial analysis, which would suggest that NR processing affects the local connectivity at low SNR differently than that of at high SNR. It should be noted that the *p*-values corresponded to the interaction at the parietal, and all frequency bands are small. However, the dominant significant change due to the interaction between SNR and NR appears to be at the alpha band as only the parietal alpha band survives a correction for multiple comparisons.

The articulation-index-weighted SNR improvements (Ohlenforst et al., 2018) of the NR processing were applied, which reduces the two factors of the experiment to only one factor SNR with values +3 dB, +8 dB, +9.24, and +13.17 dB. We then investigated the relationship between the experimental conditions and local connectivity. We found a significant inverted U-shaped function at parietal alpha by both single and average trial analysis, which was in line with our second Hypothesis H2. Since this study is the first work, to our knowledge, that investigates the effect of different levels of listening effort and NR schemes in HA on local connectivity, the results will be discussed in terms of hypothesized functions in the following sections.

### 4.2. NR schemes in HAs reduce the listening effort

Recent studies have shown that the activation of advanced signal processing algorithms in HAs provides hearing benefits for HIs, particularly in adverse listening conditions (Sarampalis et al., 2009; Ohlenforst et al., 2018; Winneke et al., 2018). Studies focusing on changes or benefits in speech intelligibility may not provide a complete picture of the processes involved in speech recognition (Dillon and Lovegrove, 1993; Sarampalis et al., 2009; Ohlenforst et al., 2018). In particular, NR processing, which is the main focus of this study, can reduce listening effort and free up cognitive resources for other tasks while it may not have positive effects on speech reception threshold (Sarampalis et al., 2009). The effects of NR schemes have been investigated when the stimuli is a single word or sentence (Dimitrijevic et al., 2017; Miles et al., 2017; Ohlenforst et al., 2018). However, HI individuals encounter long speech in real ecological situations. For this reason, long continuous news clips were presented at different SNR levels. Our first finding based on speech tracking analysis of the EEG data published in Alickovic et al. (2020) showed that NR processing can improve the performance of HAs during a selective auditory attention task. Then, Fiedler et al. (2021) showed that the NR schemes can also reduce the listening effort estimated by pupillometry. However, the neural index of listening effort estimated by spectral power analysis of the EEG data did not show any statistical change. It inspired us to recruit a new correlate of listening effort estimated by local connectivity in EEG data to investigate the effect of NR schemes during a long continuous SiN task.

As shown in Table 2A, the interaction between SNR and NR on local connectivity is statistically significant. This suggests that NR processing affects the local connectivity differently at the two SNR values of the experiment, which is in line with pupillometry results reported in Ohlenforst et al. (2018) and Fiedler et al. (2021) where they also found a different effect of NR schemes at different SNR values. This result is also consistent with results published in Alickovic et al. (2020) in which they found that NR processing improved the performance of the selective attention task differently at the two SNR values. We also investigated the relationship between correlates of listening effort and the experimental conditions by applying the SNR-improvement of NR processing. As demonstrated in Figures 3, 4 and Table 3A, we found an inverted U-shaped trend. We believe that this study is the first work that showed the nonlinear trend of neural estimation of correlates of listening effort as a result of NR processing at different SNR values during a continuous long SiN task. This result is consistent with pupil dilation analysis in Ohlenforst et al. (2018) and EEG analysis in Marsella et al. (2017), Wisniewski et al. (2017), Decruy et al. (2020), and Paul et al. (2021) where nonlinear relationship due to NR processing at different SNR values and different levels of listening difficulty were found, respectively.

### 4.3. Local connectivity is modulated by top-down cognitive functions or changes of brain networks

Most of the existing studies in the literature which investigated the listening effort by using EEG signals tend to focus on spectral power features and particularly event-related spectral perturbation (ERSP) (c.f Section 4.5). Finding a relationship between local connectivity estimated at scalp level, which is the case in this study, and power change can be controversial and even two features can be significantly uncorrelated, as is the case in Jalili et al. (2007) study where no significant correlation was found between power change and local connectivity in Schizophrenia EEG data analysis. There might be possibilities to discuss the relationship based on the Firefly model presented in Burgess (2012) or the model presented in Jirsa (2009). However, we believe that the local connectivity estimated in this study can violate the required assumptions of these models. Nonetheless, the studies which investigate spectral power changes during effortful listening described the possible top-down cognitive functions or brain networks that can lead to the change in the power features. There are two theories that can connect top-down cognitive function or brain networks and local connectivity.

The first theory is based on the phase reset model in which phase locking of ongoing EEG activity can be a modulatory effect of top-down functions of the brain (Bernarding et al., 2017). Peelle et al. (2013) found that neural data and the envelope of the external acoustic stimuli become more phase-locked when linguistic information is available. They concluded that the phase-locking of the neural oscillations does not rely only on sensory cues and top-down cognitive function can also modulate phase locking. Dimitrijevic et al. (2019) also found that the phase-locked cortical representation can be modulated by top-down cognitive function related to different levels of listening effort. Bernarding et al. (2017) demonstrated that the distribution of the phase of the ongoing EEG signal can be modulated by the top-down cognitive functions related to different listening efforts. Considering these aspects, one interpretation can be that local connectivity estimated by multivariate phase synchrony is also modulated by the top-down cognitive functions related to the listening effort.

The second theory explains that change in local connectivity estimated by phase synchronization can be one of the mechanisms for coordinating the information transfer in brain networks (Helfrich et al., 2016; Olejarczyk et al., 2017). For example, Helfrich et al. (2016) showed that local parieto-occipital phase coupling at the alpha band controls the inter-hemispheric information transfer. Additionally, Olejarczyk et al. (2017) reported an increase in local phase coupling in closed eyes compared to open eyes in a resting-state EEG analysis and they concluded that fronto-parietal information transfer can be regulated by local phase synchrony. Regarding these aspects, it can be interpreted that local connectivity estimated by phase synchrony also coordinates the information transfer related to effortful listening and NR schemes in HAs.

### 4.4. Significant change at parietal alpha local connectivity

Most prior studies that investigated EEG correlates of listening effort have tended to restrict their analysis to a single EEG band and region (Wisniewski et al., 2015; Dimitrijevic et al., 2019; Seifi Ala et al., 2020). However, Wisniewski et al. (2021) conducted a comprehensive study in which they investigated a fuller range of the EEG power spectrum and independent source activities. They found several significant changes in different regions and bands. They concluded that listening in adverse conditions can possibly engage multiple cognitive processes. Consistent with Wisniewski et al. (2021), NR processing can also engage multiple cognitive processes which can possibly change the local connectivity. As the effect of NR processing in HAs on local connectivity in ecologically adverse conditions was investigated for the first time in this article, we did not restrict our analysis to one specific EEG band and region. Local connectivity at a total of eight ROIs and four conventional EEG bands were, therefore, examined.

Frontal theta and parietal alpha activity at the sensor level have been mainly reported in the literature as the regions and bands that can be used to estimate correlates of listening effort (Wisniewski et al., 2017, 2018; Dimitrijevic et al., 2019; Fiedler et al., 2021). The change in the frontal theta activity is mostly observed in experiments in which non-speech stimuli were used (Wisniewski et al., 2017, 2018). This tends to reflect the internal attention and it does not show general endogenously exerted effort related to externally generated object representations (e.g., competing speech streams and background noise) (Wisniewski et al., 2018). As the change of listening effort in this study is mostly due to changes in externally represented speech stimuli, we did not observe any significant change in frontal theta local connectivity, which is in line with the results reported in Seifi Ala et al. (2020) that significant change of frontal theta change was not observed as a results changes in the speech stimuli characteristics. On the other hand, the change of parietal alpha activity has been widely observed when listening effort was examined in experiments with speech stimuli (Petersen et al., 2015; Wöstmann et al., 2015, 2017b; McMahon et al., 2016; Dimitrijevic et al., 2017, 2019; Marsella et al., 2017; Miles et al., 2017; Seifi Ala et al., 2020; Fiedler et al., 2021; Paul et al., 2021), which is line with our results where we only found significant change at the parietal alpha activity.

### 4.5. Top-down cognitive functions in listening effort

The direction (i.e., increase, decrease, or inverted U-shape) of the parietal alpha activity modulation found in the literature has been controversial. Some studies reported that higher listening effort leads to an increase in parietal alpha power (relative to the baseline) arguing that it reflects the inhibition of neural activity in task-irrelevant brain area (Petersen et al., 2015; Wöstmann et al., 2015, 2017b; McMahon et al., 2016; Dimitrijevic et al., 2017, 2019; Marsella et al., 2017; Miles et al., 2017; Paul et al., 2021). In contrast, other studies showed that more demanding conditions lead to a decrease in parietal alpha power (Seifi Ala et al., 2020; Fiedler et al., 2021). The first explanation for the contradictory results is that multiple sources of alpha power contribute to parietal alpha power and the balance between suppression and enhancement can be determined by the stimuli and task design (Dimitrijevic et al., 2017; Seifi Ala et al., 2020; Fiedler et al., 2021; Wisniewski et al., 2021). Seifi Ala et al. (2020) observed lower parietal alpha power related to more difficult conditions during long speech listening. It was discussed in Seifi Ala et al. (2020) that sustained attention and constant update of information in working memory is required when the stimuli are long, which would lead to contradictory results. Another explanation for the opposite direction can be related to the inverted U-shape relationship. Depending on the level of difficulties of the experiment, estimated correlates of listening effort can be on one or the other side of the inverted U's maximum, which would result in an increase or decrease in parietal alpha power, respectively (Fiedler et al., 2021).

The last relationship between listening conditions and parietal alpha power reported in the literature is an inverted U-shape (Marsella et al., 2017; Wisniewski et al., 2017; Decruy et al., 2020; Paul et al., 2021). There are two explanations for the observed nonlinear trend. One theory is that during difficult conditions subjects disengaged and gave up to perform the task, which can influence the parietal alpha changes (Marsella et al., 2017). The second explanation is that at very high levels of difficulty, other sensory networks might be activated to help speech understanding which leads to an inverse direction of parietal alpha modulation compared to that at lower difficulty levels (Paul et al., 2021). The supportive sensory networks under very hard conditions can be related to sustained attention and constant update of information in working memory as reported in Seifi Ala et al. (2020).

### 4.6. Parietal alpha local connectivity is modulated by listening effort

Referring to Figure 3 and Table 3A, we also found a significant nonlinear trend in local connectivity at parietal alpha. As shown in Figure 3, an increase in levels of difficulty in listening (decrease in SNR values) from the condition +8 dB ON (NR: active) to +8 dB off (NR: inactive) leads to an increase in local connectivity. Consistent with results reported in Petersen et al. (2015), Wöstmann et al. (2015, 2017b), McMahon et al. (2016), Dimitrijevic et al. (2017), Marsella et al. (2017), Miles et al. (2017), Dimitrijevic et al. (2019) and Paul et al. (2021), the increase of the parietal alpha power can be due to inhibition cognitive function. Considering the first theory mentioned in Section 4.3, the inhibition of top-down cognitive function can lead to the modulation of local connectivity. This interpretation is in line with the results of Mathewson et al. (2009) and Paul et al. (2021) where the authors also found that phase synchronization in parietal alpha increases due to inhibition of cognitive function. The change of local connectivity due to inhibition function can also be supported by the second theory mentioned in Section 4.3. The inhibition function mostly engages the fronto-parietal network. We interpreted that the local connectivity at parietal alpha can also coordinate the fronto-parietal information transfer. This interpretation is in line with the results reported in Olejarczyk et al. (2017) where the authors also found that phase synchrony in parietal alpha coordinates the fronto-parietal information transfer in rest-state EEG analysis.

As shown in Figure 3, in the more difficult condition (i.e., at +3 dB) the local connectivity decreases, whereas in the easier condition (i.e., at + 8dB) the local connectivity increases. In line with EEG band power analysis results, this change can be due to either giving up during more difficult conditions (Marsella et al., 2017) or other sensory networks that might be activated to help speech understanding during such listening conditions (Paul et al., 2021). Our findings provide evidence that the change from increase to decrease in local connectivity under more difficult conditions could be due to the activation of other networks at the lowest SNR value. Considering the second theory mentioned in Section 4.3 which describes that local connectivity estimated by phase synchrony can coordinate the information transfer in brain networks, the change of direction in local connectivity modulation at +3 dB OFF condition can also be due to activation of other sensory networks which can be coordinated by local connectivity at parietal alpha. One possible sensory network can be due to sustained attention and constant update of information in working memory which is in line with the results reported in Seifi Ala et al. (2020). It was discussed in Seifi Ala et al. (2020) that sustained attention and constant update of information in working memory are required when the stimuli are long. This was also observed during a Stenberg task in which encoding and retention phases were entangled and a contradictory increase in parietal alpha power was reported as a result of higher working memory loads (Jensen et al., 2002; Hjortkjær et al., 2020; Seifi Ala et al., 2020). Kim et al. found that the brain network involved in updating function engaged in an n-back level experimental paradigm mostly includes the parietal cortex which is served as the main hub for the cognitive network (Kim et al., 2017). They also found a substantially different pattern during the most demanding condition compared to easier conditions. Considering the second theory in Section 4.3, the change of direction of the local connectivity modulation at the hardest condition in our experiment can also be due to substantially different networks involved in updating function.

### 4.7. Limitations

The local connectivity at eight ROIs and four EEG frequency bands were investigated in this study. The selection of the ROIs was similar to that in Mehta and Kliewer (2017) where they used 128 electrodes, and we adapted their ROI selections with 64 electrodes setup. However, there might be a better selection of ROIs, which can lead to different results. Additionally, considering that we had two states of NR processing and two SNR values, our experiment had four listening conditions. We checked the relationship between local connectivity and listening conditions, and we found a nonlinear trend. Examination with more SNR values is required, which can provide more insights, and we expect to observe a complete inverted U-shape relationship with more SNR values.

Obleser and Kayser (2019) showed that the phase locking between neural data and the envelope of the speech can be modulated by the behavioral performance of the task. There is a possibility that local connectivity is also modulated by the performance of the task or subjective rating of listening effort (often referred to as self-report or experienced listening effort) (Paul et al., 2021), similar to the first theory in Section 4.3. The behavioral performance evaluation was accomplished by asking a two-choice question about the to-be-attended speech stream at the end of each trial in our experiment. Our investigation of the effect of the experimental factors on behavioral performance published in Fiedler et al. (2021) did not show any significant effect of NR, SNR, or their interactions on the behavioral performance. The behavioral performance was though well above the chance level (50%) and the participants followed the task as instructed. However, the lack of valuable behavioral performance or subjective ratings of listening effort prevented us from checking the possibility that local connectivity is modulated by them.

The second theory in Section 4.3 explained that local connectivity can coordinate the information transfer in brain networks. We interpreted that local connectivity at parietal alpha can also coordinate the large-scale connectivity engaged in inhibition function and constant update of the working memory and referred to the studies in which these information transfers were studied. There is a possibility that other brain networks are also engaged during a continuous long SiN task, which could be provided by a large-scale connectivity investigation.

## 5. Conclusion

We investigated the effect of activation of NR processing on EEG-based phase synchrony measure within localized regions of the brain at eight regions of interest and four conventional EEG frequency bands during a longer continuous speech in noise (SiN) task with two SNR levels. We demonstrated that the effect of noise reduction (NR) processing algorithms on EEG-based phase synchrony have a non-linear trend in the parietal region of interest, specifically in the alpha band. The interpretation of the phase synchrony modulation is in line with the literature. These results confirmed that the EEG-based phase synchrony within localized regions of the brain contains informative features which can reflect the effects of HA signal processing algorithms in HA users. Taken together, our study provided further evidence that the NR processing algorithms in HAs positively affect HA users in their everyday natural listening environments.

## Data availability statement

MATLAB and R codes of this article can be found in the Supplementary material, further inquiries can be directed to the corresponding author/s.

## Ethics statement

The studies involving human participants were reviewed and approved by Ethics Committee for the capital region of Denmark (journal number H-1-2011-033). The patients/participants provided their written informed consent to participate in this study.

## Author contributions

PS, CG, EA, and JØ contributed to conception and design of the study. EA organized the database. PS performed the software and statistical analysis and wrote the first draft of the manuscript. All authors contributed to manuscript revision, read, and approved the submitted version.

## Conflict of interest

The authors declare that the research was conducted in the absence of any commercial or financial relationships that could be construed as a potential conflict of interest.

## Publisher's note

All claims expressed in this article are solely those of the authors and do not necessarily represent those of their affiliated organizations, or those of the publisher, the editors and the reviewers. Any product that may be evaluated in this article, or claim that may be made by its manufacturer, is not guaranteed or endorsed by the publisher.

## Footnotes

^1^An alternative group of electrodes for frontal, central, parietal, and occipital ROIs, which include the midline electrodes located in the regions, such as Fz, Pz, Cz, and Oz produced the same trend of results.

^2^An alternative longer length (such as 10 s) of time windows leads to a better estimation of COC. However, the results produced by longer or equal to 2 s time windows were following the same trend.
